# Different Outcomes of “Silent Hypoxia” in Patients with COVID-19 Pneumonia: A Case Series and Literature Review

**DOI:** 10.1155/2021/1215274

**Published:** 2021-09-03

**Authors:** Ashani Ratnayake, Prabhashini Kumarihamy, Sujeewa Gunaratne, Hiranya Abeysinghe, Sahan Perera, Shirley Ekanayake

**Affiliations:** District Base Hospital, Teldeniya, Sri Lanka

## Abstract

COVID-19 has been declared a pandemic since March 2020 and it has been responsible for millions of deaths worldwide. The SARS-CoV-2 causes a spectrum of diseases mainly affecting the respiratory system. It can also complicate other systems causing thromboembolic phenomena and myocardial ischaemia. An entity of hypoxia has been described in these patients which show no clinical signs and symptoms of respiratory distress despite being extremely hypoxic. This is called silent or happy hypoxia. The exact mechanism for this is not known. We report 4 cases which had similar presentations of silent hypoxia but had different course of illness and different outcomes. All 4 patients did not show any signs of respiratory distress, but had oxygen saturation less than 82%. 3 of them needed intensive care unit support for oxygen therapy and subsequently needed noninvasive ventilation. Only one required invasive ventilation. The fourth patient did not require intensive care support. The patient who required invasive ventilation succumbed due to severe COVID pneumonia whereas the other 3 patients were discharged from the hospital. Silent hypoxemia can go undetected in COVID-19 patients particularly in the time of a pandemic. This case series highlights the importance of meticulous clinical examination including oxygen saturation measurements in suspected or confirmed patients with COVID-19. The course of illness can be different in different populations, and this needs further clinical evidence.

## 1. Introduction

SARS-CoV-2 virus is responsible for the spectrum of conditions in COVID-19. It is predominantly a respiratory infection that can complicate other systems. It may present with a wide spectrum of symptoms ranging from mild self-limiting upper respiratory tract infection like illness to severe pneumonia and death. The SARS-CoV-2 infection leads to severe respiratory failure in some of the patients who require advanced respiratory support. Often deterioration to this level is evident by clinically detectable respiratory distress as dyspnoea or tachypnoea. “Silent” or “apathetic” hypoxia is an entity described in patients suffering from COVID-19 pneumonia which is described as objective hypoxia in the absence of apparent respiratory distress [1, 2].

There is a certain group of patients who presents with silent hypoxia. Those patients lack proportionate clinical signs of respiratory distress, even though their chest X-rays show diffuse pneumonia and have evidence of hypoxia detectable in pulse oximetry or arterial blood gas analysis. However, sudden and rapid deterioration may occur in this subset of patients. To assess the severity of pulmonary involvement, a clinical examination should be meticulous, and adequate emphasis should be given to pulse oximetry reading and blood gas analysis. The cause of silent hypoxaemia is controversial. The outcome of these patients can also be variable, and no predictors have been identified to comment on the prognosis. We report 4 cases with silent hypoxaemia with the same presentation, but a different course of illness. The purpose of this report is to highlight the importance of “silent hypoxia” in COVID-19 patients.

## 2. Case 1

**Figure 1:**
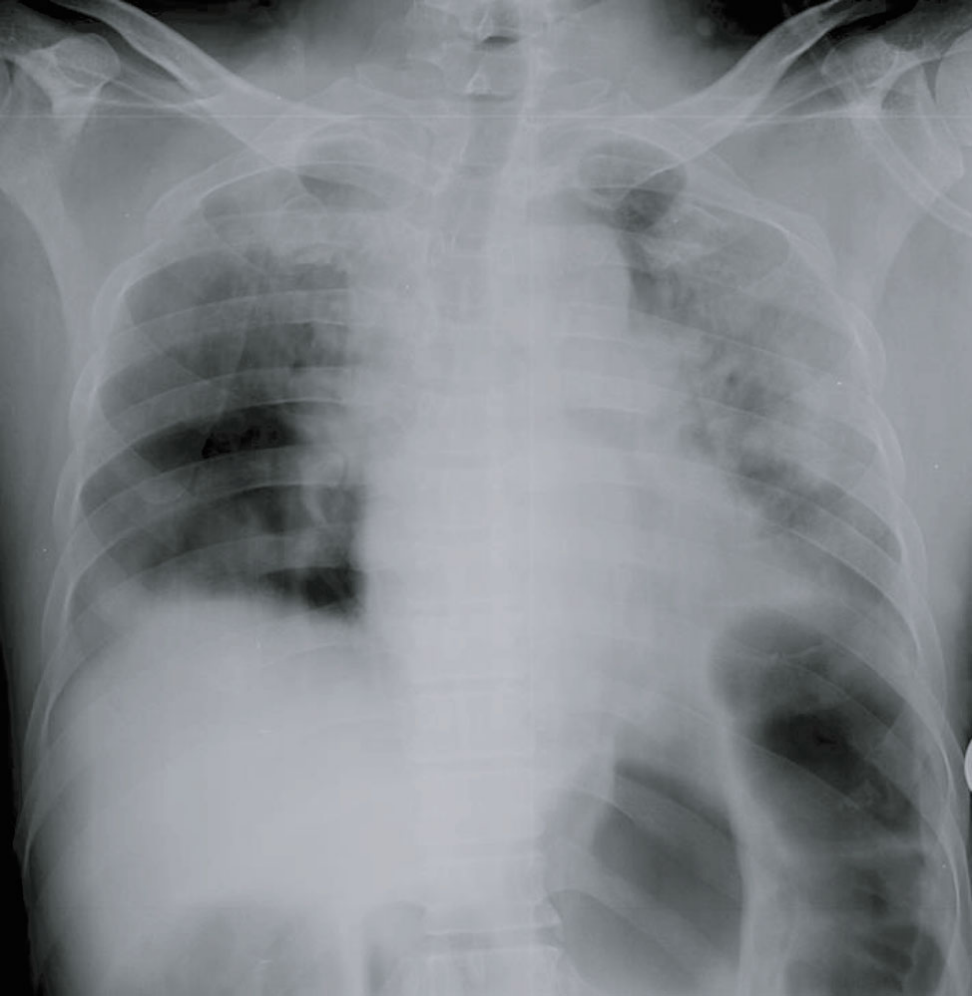
Chest radiograph of case 1 on the day of presentation with silent hypoxaemia showing extensive air space opacification involving both lungs with more changes on the left side.

**Figure 2:**
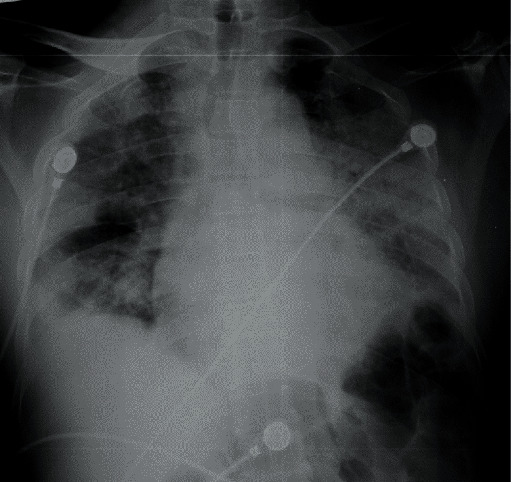
Chest radiograph of case 1 on day 3 of ICU showing progression of air space opacification in both lungs.

A 53-year-old manual labourer with a history of fever, productive cough, and sore throat for 3 days presented to a local hospital. He is a current smoker and did not have any comorbid diseases. He was diagnosed with a COVID-19 infection by a rapid antigen test and was transferred to a specialised COVID-19 treatment centre. He was not in acute distress and conversant with unlabored speech. His respiratory rate was 16/min without any dyspnoea. He had a blood pressure of 134/77 mmHg with a heart rate of 88/min. On pulse oximetry measurement, his saturation was found to be 66% on room air, and arterial oxygen partial pressure was 56 mmHg. His CXR showed bilateral patchy consolidations on admissions (Figure 1). He was admitted to the intensive care unit for oxygen therapy and required 60% O2 via a face mask. In the next 2 days, his hypoxia worsened requiring noninvasive ventilation (NIV). Subsequent chest X-rays showed gradual worsening of the air space shadowing (Figure 2). He received IV dexamethasone 6 mg daily and subcutaneous enoxaparin. He was on NIV for 48 hours which was subsequently weaned on to high flow nasal oxygen (HFNO). This was weaned off over the next 5 days. His blood investigations are given in Table 1. He had a total ICU stay for 16 days requiring intermittent oxygen therapy. Further, he had multiple episodes of silent hypoxaemia at the time of weaning from respiratory support. On discharge from ICU to ward, his saturation on room air was 90%. He was discharged from the hospital after 21 days.

## 3. Case 2

**Figure 3:**
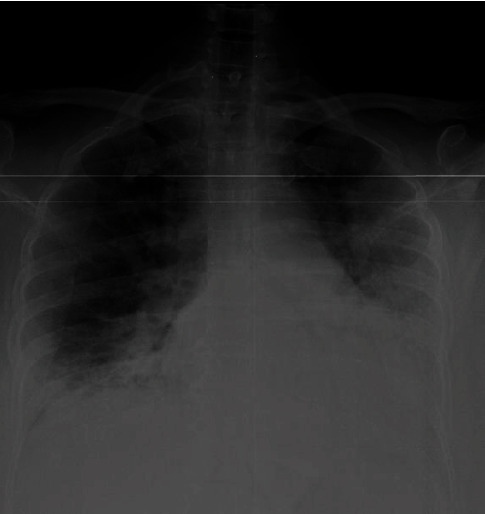
Chest radiograph of case 2 on the day of admission with silent hypoxaemia showing bilateral lower zonal opacification. There is more involvement on the left side.

**Figure 4:**
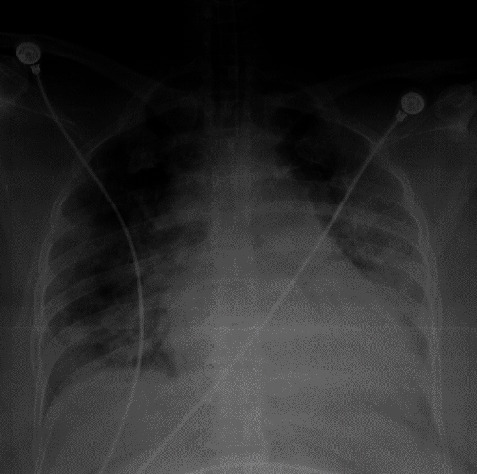
Chest X-ray of case 2 after 3 days in ICU. Endotracheal tube in situ. There is worsening of the shadowing of both lungs.

A 51-year-old lady with a background history of diabetes mellitus and hypertension presented to a local hospital with cough and fever for 7 days duration. She had a BMI of 39. She was transferred to a specialised COVID treatment centre after tested positive for SARS-CoV-2 rapid antigen. She denied any dyspnoea or chest pain on admission. Her respiratory rate was 20/min with a blood pressure of 155/80 mmHg and a pulse rate of 77/min. Despite the absence of signs of respiratory distress, her saturation on admission was 60% on air. Arterial blood gas revealed an arterial oxygen saturation of 51 mmHg with 26 mmHg of CO2 on room air. Her chest X-ray showed bilateral patchy consolidation (Figure 3). She was transferred to ICU for oxygen therapy. She received NIV for the first 24 hours where she became increasingly hypoxemic requiring intubation and invasive ventilation. Repeated chest X-ray showed further deterioration (Figure 4). She had a high oxygen demand ranging from 80 to 100% of oxygen and high PEEP with mechanical ventilation. She was initially on IV dexamethasone 6 mg, and after 4 doses, this was converted to IV methylprednisolone 1 g pulse therapy and continued for 3 days. Her laboratory investigations are illustrated in Table 1. She became increasingly hypoxemic and was offered prone ventilation. Careful positioning was necessary as she had a high BMI. Despite all ventilatory strategies, she succumbed on day 8 of ICU stay.

## 4. Case 3

**Figure 5:**
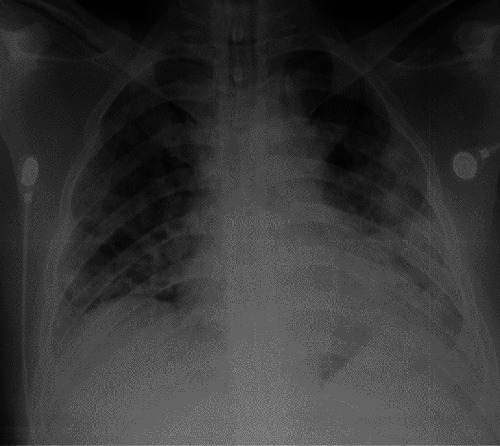
Chest radiograph of case 3 on day of the presentation with silent hypoxaemia showing widespread air space opacification in both lungs predominantly involving the lower zones.

**Figure 6:**
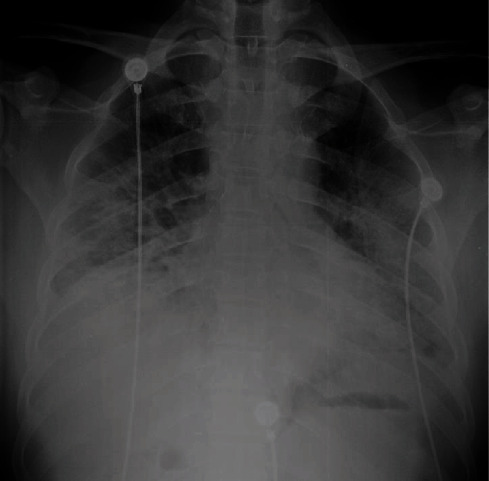
Chest radiograph of case 3 on day 3 of admission to ICU showing worsening of air space opacification in both lungs. More changes are seen on the left lung.

A 43-year-old gentleman with well-controlled diabetes, ischaemic heart disease with history of PCI to LAD 2 years back was diagnosed with COVID-19 infection and transferred to specialized COVID-19 treatment centre due to his complicated medical history. He was on clopidogrel, aspirin, and bisoprolol. On arrival, he complained of fever and cough for 2 days with a sore throat. He denied any difficulty in breathing and was not dyspnoeic at rest or on exertion. He had a blood pressure of 140/73 mmHg with a pulse rate of 68/min. On admission pulse oximetry showed a saturation of 68% on room air with a PaO_2_ of 54 mmHg. He had a respiratory rate of 16/min and initial CXR showed bilateral patchy consolidations (Figure 5). The patient was sent to ICU, and high flow nasal oxygen therapy commenced with FiO2 of 70% and a flow rate of 60 L/min. He was started on IV dexamethasone 6 mg daily and continued for 10 days. The laboratory investigations are shown in Table 1. His condition deteriorated over the next 6 days and on day 6 on ICU, noninvasive ventilation was commenced. Chest X ray showed deterioration of air space opacification (Figure 6). He was treated with a single dose of IV tocilizumab 400 mg. The patients showed a rapid response to IV tocilizumab, and from day 7 onwards, the oxygen requirement was gradually declining. He was discharged on day 14 from ICU and day 19 from the hospital. He did not have any complications during the stay.

## 5. Case 4

**Figure 7:**
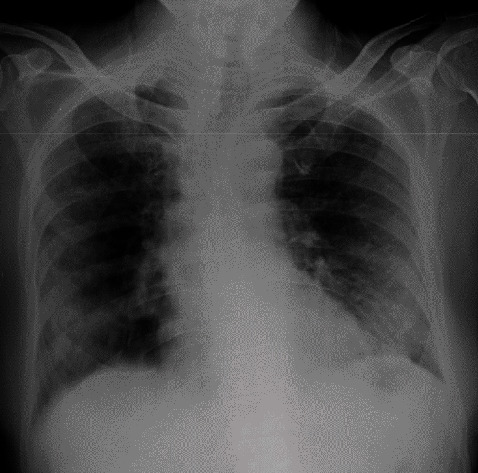
A chest radiograph of case 4 at the time of presentation with silent hypoxia showing bilateral air space opacification.

**Figure 8:**
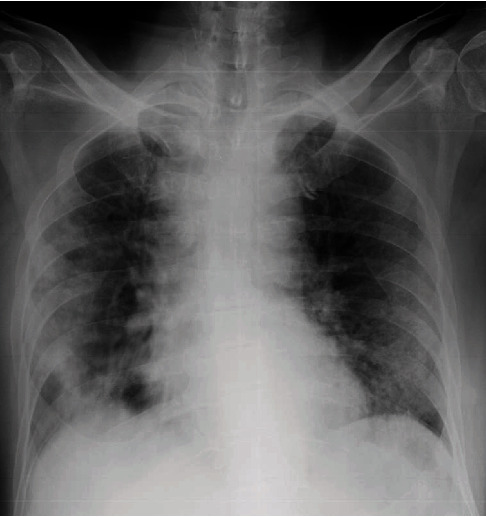
Chest radiograph of case 4 on day 2 in the ICU showing worsening of air space opacification.

A 60-year-old male with a background history of diabetes, hypertension, and ischaemic heart disease presented to the local hospital with fever, cough, and sore throat for four days. He was found to be positive for SARS-CoV-2 and was transferred to our centre for specialized COVID management. On admission, he had oxygen saturation (SpO2) of 82% on air with PaO2 of 56 mmHg with normal respiratory rate and effort. His blood pressure was 160/80, and his pulse rate was 90/min. His oxygen saturation improved to 98% on FiO2 of 40%. His CXR showed bilateral air space opacification (Figure 7). A chest X-ray repeated 2 days later showed worsening of these findings (Figure 8). He was dependent on 40% oxygen in the initial 4 days, and then, the oxygen was weaned off. He did not require admission to ICU. Laboratory findings are illustrated in Table 1. He was discharged home after 16 days of hospital stay.

## 6. Discussion

SARS-CoV-2 is a droplet borne virus responsible for the global pandemic of COVID-19 infection. Millions of cases have been reported worldwide. The disease spectrum ranges from asymptomatic infection to acute respiratory distress syndrome causing death in a considerable number of patients. The course of infection can also complicate with thromboembolic phenomena, myocardial ischaemia, and secondary infections [3, 4].

Hypoxaemia is defined as arterial oxygen partial pressure less than 80 mmHg, and it becomes severe when it falls below 60 mmHg [5]. Hypoxaemia often accompanies signs and symptoms of respiratory distress, namely, tachypnoea and dyspnoea. In SARS-CoV-2 infection, some patients lack these clinical signs and symptoms of hypoxaemia despite alarmingly arterial oxygenation. This can only be detected by objective measurements like pulse oximetry readings and arterial blood gas measurements. This entity is called silent hypoxaemia or happy hypoxaemia, and this can be detrimental in some patients [1, 2]. Chauhan et al., hypothesized silent hypoxemia which is occurring at the initial stages of the infection, and if it is undetected and unaddressed, this persistent hypoxia contributes to a complex and vicious cycle of increased coagulopathy, cytokine storm, and ultimately more hypoxia/hypoxemia.

The exact mechanism for silent hypoxaemia is not clear. But there are several hypotheses to explain the occurrence of this entity. Most of the hypothesis is related to the preservation of the ability to remove CO2 from the body during COVID-19 infection. Pneumonia analysis of COVID-19 shows the virus causes the alveoli to collapse, reducing the oxygen level and causing hypoxia, but the reaction still enhances the normal lung ability to expel carbon dioxide. Since carbon dioxide removal is still effective, patients do not feel shortness of breath [6]. It is also proposed that intrapulmonary shunting can contribute to this. The infection causes interstitial oedema, loss of surfactant, and superimposed pressure, which induces alveolar collapse and a significant fraction of cardiac output perfusing nonaerated lung tissue. With time, increased oedema will cause alveolar collapse and dependent atelectasis, leading to increased shunting still preserving the ability to remove CO2 [7].

There are not enough data available to predict the clinical course or outcome in these patients with silent hypoxaemia. Chandra et al. reported a case of a 56-year-old patient who presented with silent hypoxemia who made complete recovery only with supplemental oxygen up to 15 L/min via a nonrebreather mask. He did not require ventilator support [8]. Lari et al. reported another case of a 66-year-old patient who had silent hypoxemia who eventually needed high flow oxygen therapy and NIV [9]. Similar to our first patient, this patient had also shown multiple episodes of silent hypoxemia at the time of weaning from oxygen therapy. The case reported by Siswanto et al. had a worse outcome. This was a 60-year-old lady with silent hypoxaemia who needed invasive ventilation and succumbed during ICU stay [5]. Our second patient had a similar outcome.

We have reported 4 cases that we encountered who had the clinical entity of silent hypoxaemia. None of them showed apparent signs and symptoms of respiratory distress despite having alarmingly low SpO_2_ level and diffuse pneumonia in CXR. Once the patients were diagnosed to have silent ischaemia, they were started on oxygen therapy. Case 1, 2, and 3 patients showed an increment in their oxygen requirement with disease progression following the diagnosis of silent hypoxaemia. In contrast the 4^th^ patient remained on the same requirement and subsequently weaned off without the need for the ICU care. Therefore the course of illness is not similar in all patients with silent hypoxaemia. The outcome of patients was also different. Case 1 had prolonged hospital stay, and the recovery was complicated with several episodes of silent hypoxaemia while 2^nd^ patient died after getting ARDS. The 4^th^ patient was discharged without any complications.

All 4 patients received IV dexamethasone as a part of their treatment regimen. The third patients received IV tocilizumab when he was deteriorating rapidly. Use of tocilizumab can be commenced in hospitalized patients with COVID-19 in whom there are no signs of infection [10]. The evidence was new at the time of management of case 3, and rest of the patients were not given it as they were managed before the publication of guidelines.

Silent hypoxemia is an important entity which needs to be detected early in patients with SARS-CoV-2 infection. However, the course of illness could be unpredictable. The exact cause of the silent hypoxaemia is also not known. In our experience, further clinical evidence is required to interpret the associations and outcome of silent hypoxemia.

## 7. Conclusions

Silent hypoxemia can go undetected in COVID-19 patients. This highlights the importance of meticulous clinical examination including oxygen saturation measurements in suspected or confirmed patients with COVID-19. The course of illness can be different in different populations, and this needs further clinical evidence.

## Figures and Tables

**Table 1:** Laboratory investigations of the patients presented with silent hypoxaemia.

	Case 1			Case 2			Case 3			Case 4		
	Day 1	Day 5	Day 10	Day 1	Day 5	Day 10	Day 1	Day 5	Day 10	Day 1	Day 5	Day 10
WBC count (×10^9^/L)	18.66	13.70	11.40	12.40	11.80	N/A	13.40	12.40	11.20	9.50	9.91	10.40
*N* (×10^9^/L)	17.28	11.54	10.20	11.60	10.20	N/A	11.30	10.30	11.70	8.73	10.5	9.10
*L* (×10^9^/L)	0.63	1.72	1.16	1.69	1.45	N/A	0.43	1.67	1.56	0.37	1.54	1.50
*E* (×10^9^/L)	0.04	0.03	0.13	0.03	0.04	N/A	0.04	0.05	0.03	0.04	0.20	0.30
Hb (g/dL)	12.80	12.10	13.70	10.90	8.10	N/A	13.4	14.40	14.10	11.50	11.80	11.9
Platelets (×109/mL)	289	394	231	384	247	N/A	342	356	332	315	399	366
CRP (mg/L)	190	33.60	N/A	268	162	N/A	223	112	68	31	3.34	1.80
SGOT (units/L)	16.60	20.80	21	23	88.20	N/A	35.50	31.20	32.10	35.90	37.20	N/A
SGPT (units/L)	28	22.20	20.10	15.70	110.6	N/A	32.30	30.40	30.20	37.80	23.70	N/A
Bilirubin-total (mg/dL)	0.63	0.90	0.74	0.22	0.43	N/A	0.61	N/A	0.65	0.42	N/A	N/A
Bilirubin-direct (mg/dL)	0.12	0.15	0.44	0.05	0.20	N/A	0.15	N/A	0.76	0.16	N/A	N/A
Serum creatinine (mg/dL)	1.11	1.00	1.04	0.72	1.29	N/A	0.90	0.98	0.95	9.50	0.95	N/A
Blood urea (mg/dL)	66.90	38.10	39.70	53.40	74.50	N/A	56.30	55.10	45.20	62.20	N/A	N/A
Procalcitonin (ng/mL)	3.20	N/A	N/A	0.42	1.57	N/A	0.49	0.34	N/A	0.08	N/A	N/A

*N*: *B*: day 1 is the day the patient was diagnosed with silent hypoxia. Case 2 did not survive 10 days; therefore, day 10 investigations were not available. N.A.: not available.

## Data Availability

All data relevant to the case series are given in the report submitted. Additional data can be submitted on request.

## References

[1] Widysanto A., Wahyuni T. D., Simanjuntak L. H. (2020). Happy hypoxia in critical COVID-19 patient: a case report in Tangerang, Indonesia. *Physiological Reports*.

[2] Ottestad W., Seim M., Mæhlen J. O. (2020). Covid-19 med stille hypoksemi. *Tidsskr den Nor Laegeforening*.

[3] Chauhan A., Kaur R., Chakrbarti P., Pal A. (2021). “Silent hypoxemia” leads to vicious cycle of infection, coagulopathy and cytokine storm in COVID-19: can prophylactic oxygen therapy prevent it?. *Indian Journal of Clinical Biochemistry*.

[4] Srivastava A. (2020). Blood clots in the lung may be a major cause of COVID-19 deaths. *The Hindu*.

[5] Siswanto, Gani M., Fauzi A. R. (2020). Possible silent hypoxemia in a COVID-19 patient: a case report. *Annals of Medicine and Surgery*.

[6] Teo J. (2020). Early detection of silent hypoxia in Covid-19 pneumonia using smartphone pulse oximetry. *Journal of Medical Systems*.

[7] Dhont S., Derom E., Van Braeckel E., Depuydt P., Lambrecht B. N. (2020). The pathophysiology of “happy” hypoxemia in COVID-19. *Respiratory Research*.

[8] Chandra A., Chakraborty U., Pal J., Karmakar P. (2020). Silent hypoxia: a frequently overlooked clinical entity in patients with COVID-19. *BMJ Case Reports*.

[9] Lari A., Alherz M., Nouri A., Botras L., Taqi S. (2020). Caution against precaution: a case report on silent hypoxia in COVID-19. *BMJ Case Reports*.

[10] National institute of healthcare and Excellence (2021). COVID-19 rapid guideline managing COVID-19 [NICE guidelines No191]. https://www.nice.org.uk/guidance/ng191/resources/covid19-rapid-guideline-managing-covid19-pdf-51035553326.

